# Hygiene of the Mouth

**Published:** 1895-01

**Authors:** M. H. Cryer

**Affiliations:** Lecturer on Oral Surgery in the Medico-Chirurgical Philadelphia Hospital


### Hygiene of the Mouth.

BY M. H. CRYER, M.D., D.D.S.,

Lecturer on Oral Surgery in the Medico-Chirurgical Philadelphia Hospital.

The hygiene of the mouth is a department of stomatology, the importance of which is not sufficiently appreciated. As the mouth is the portal to the alimentary canal, and one of the avenues to the lungs, it is necessary for the general welfare of the whole body that it should be kept in as healthy a condition as possible. It has been demonstrated beyond all doubt that infectious materials from an unhealthy or septic mouth find their way into the general system by being carried to the stomach or the lungs, thus causing a variety of local and general disturbances. The mouth also being concerned in taste, vocalization and mastication, it is necessary in order to properly perform all these functions, that it shall be free from foul matter, whether in the shape of retained food between the teeth, discharges from diseased gums, and alveolar processes or decayed teeth. The teeth should occupy their normal position, and be free from soreness. An unsanitary condition of the mouth is not one incompatible with normal function and sound teeth, but it is absolutely disquieting and unpardonable from an æsthetic standpoint. It stands to reason then that hygienic treatment of the mouth should begin at birth and extend throughout life. Other things being equal, the teeth should be as durable as any other tissue of the body, but the artificial methods of modern life materially lessen the resistance of these important organs. Every one, however, must recognize the importance of having them as strong and perfect as possible. The mother, by intelligent forethought, can to a great extent by eating such foods as tend to nourish and make bone and muscular tissue, give to the child before birth such nourishment as will conduce to the stronger development of the teeth and to a vigorous, healthy body. After birth, if the child receive its nourishment from the mother, she should still give the same attention to her diet. Fermentation of residual portions of milk remaining in the mouth of the infant after feeding is a prolific source of gastric curd, intestinal irritation, giving rise to colic and diarrhœa. The mouth of the child should, therefore, be kept carefully cleansed, and before each feeding be washed out with a solution of boracic acid in distilled water applied on a soft linen rag. The breast of the mother should also be carefully washed with soap and water, and also with boracic acid solution to sterilize it of fermenting films of milk which ordinarily exude from the nipple when the chest is full, as it is just before feeding time. If the child is a "bottle baby" no one but the mother or a reliable nurse should have the supervision of the bottles. The use of the long tube is unadvisable, as it requires more suction to draw the milk to the mouth, and unless a new tube were used every day cannot be kept aseptic. The black rubber nipple is the best. The bottles and nipples should be several in number—the bottle of a shape that can be easily cleaned; it is well after washing the nipples to place them in water and let them remain until they are wanted. The bottle and nipple should be removed immediately after the child has taken all it desires, as the detrimental habit of sucking a foreign substance is soon acquired and should not be allowed. The sucking power of the child is to a great extent produced by the muscles at the posterior portion of the mouth; if this action is carried on to a great extent it has a tendency to contract the width of the mouth and cause protrusion of the teeth. During the eruption of the first teeth the mouth should be frequently examined, particularly when the child shows any signs of indigestion or febrile symptoms. After fifteen years of dispensary and clinical services in the Hospital of Oral Surgery and Oral Department of the Medico-Chirurgical Hospital, it is my experience that one-half the disorders in infants from five to thirty months are caused by the abnormal eruption of the temporary teeth,and that a liberal use of the lancet, by an experienced dentist or doctor, thoroughly dividing the inflamed gum over the tooth or teeth next in order of eruption is the only rational and certain means of relief. The popular belief that the scar tissue, formed by the healing of the gum after lancing is tougher and more difficult for the tooth to penetrate, is altogether erroneous; cicatricial tissue is always more easily broken through than the normal. If the gum should heal and the same symptoms present themselves, the lancet should be used again, and the operation continued at proper intervals until the tooth is fully erupted. It is not the pressure of the tooth against the surface of the gum which causes the disturbances; the seat of the trouble is in the developing ends of the roots impinging the dental branches of the fifth pair of nerves, so that when the lancet cuts the gum until the crown of the tooth is liberated, as should always be done, the pressure upon the nerves is removed. Mothers are strangely neglectful of the teeth of their children; they will give attention to dirty hands or face and bathe the body daily, but how few keep the mouth as clean. The teeth should be gently and thoroughly brushed with a small soft tooth brush, as soon as they make their appearance, and by commencing early the child will become accustomed to its use, and even object to an omission. If better care were taken of the first set of teeth, the second would present fewer irregularities. It is a great mistake to remove deciduous teeth too soon, they should be filled if necessary and everything done to preserve them until the others are ready to take their places. By using discretion in filling the teeth with some temporary material or one that the child will bear, children need seldom be hurt. It is advisable to make the operations as simple as possible in order to avoid the dread of pain and so preventing frequent consultation with the dentist.

As the permanent teeth are erupted they should be carefully watched for irregularities, malposition, etc., as the direction of the crowns can then be changed if necessary before the teeth become thoroughly fixed in the jaw. This is an important factor in the hygienic care of the mouth. If the teeth do not articulate correctly, it will be impossible to properly masticate food, and the slighting of this important step in the great function of digestion often entails much suffering and disorder of the entire system. Perfect cleanliness, enunciation, and vocalization are also dependent upon correct position and articulation of the teeth. Decay should be prevented as far as possible, but when it occurs should receive immediate attention; the cavities should be filled in a judicious manner, with consideration for the appearance, as well as with the view of making the dental arch as perfect and self-cleaning as possible. In a great majority of cases gold should be the filling material used, as it presents a more pleasing appearance; it is cleaner, and in general it is the most durable material for the purpose. When the teeth are lost they should be replaced by artificial dentures. This again, requires good judgment on the part of the dentist, with a view of obtaining good vocalization, proper mastication, and preserving the perfect contour on the face. In using either plate or bridge for supplying the deficiencies, care must be exercised that no undue injury is done to the other teeth, and that the mouth may be as easily cleaned as before. As a rule all artificial appliances, except the simple pivot tooth and crowns, should be so made that they can be removed from the mouth and cleaned. Gold should form the basis of all such dentures. Where mouths have not had proper care in childhood, it is a somewhat difficult task to put them in a true hygienic condition. All decayed roots that cannot be made useful for attaching artificial crowns should be extracted, pulpless teeth should be treated and filled, all other cavities filled, tartar removed, and the teeth thoroughly cleaned by a competent dentist. The gums and alveolar processes should have attention either by local or systemic treatment. Instrumentation can do a great deal toward a cure, but many diseased gums and processes are caused by gouty tendencies or other systemic disorders, and they must be treated accordingly. Abscesses of the alveolar process must be cured. The bacterial condition of the mouth must be carefully considered. All of these matters should receive attention before vacant places are supplied by artificial appliances. After the mouth has been put in good condition the patient should be instructed how to keep it so. The kind of tooth brushes, as well as tooth powders and washes, should vary according to the character of the mouth and general health of the system.

Tooth brushes should be of medium size, the bristles of even length and not too stiff. In using the brush it should be carried along the cutting edges and grinding faces of the teeth with a backward and forward movement; then over the outer and inner surfaces, brushing away from the gums as much as possible. This will usually remove any particles of food, debris, etc., which have lodged between the teeth. All that cannot be removed by the brush should be taken away with a quill toothpick and by passing floss silk between the teeth. Care should be taken not to wound the margins of the gums with the brush or other appliances. The principal cleaning should be in the morning after the mouth has been in comparative rest. A slight brushing after meals and before retiring is also beneficial.

Powders which contain sharp, gritty substances should be avoided. Precipitated chalk should form the basis of all tooth powders, slightly flavored to suit the patient; other ingredients should be added according to the conditions of the secretions of the mouth.

If the mucous secretions are viscid or fetid, a wash of which lime water forms the basis will be of great service.

